# Supplementary material for: Structural basis of the molecular ruler mechanism of a bacterial glycosyltransferase
Source: Nat Commun. 2018 Jan 31;9:445. doi: 10.1038/s41467-018-02880-2 (PMC5792488; doi:10.1038/s41467-018-02880-2)
Supplement: Supplementary file 1 — Supplementary Information [file 41467_2018_2880_MOESM1_ESM.pdf]

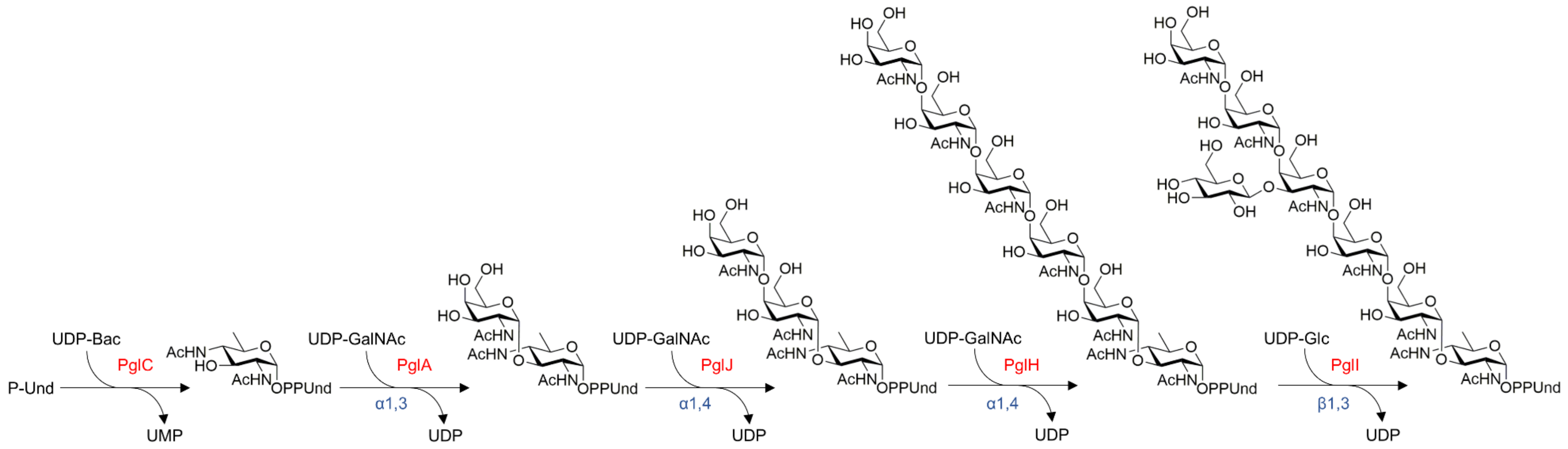

Supplementary Figure 1: Biosynthetic pathway for the biosynthesis of lipid-linked oligosaccharide in *C. jejuni*. For each step, the enzyme catalyzing the reaction is shown above the arrow and the nature of the carbohydrate linkage formed is shown below the arrow.

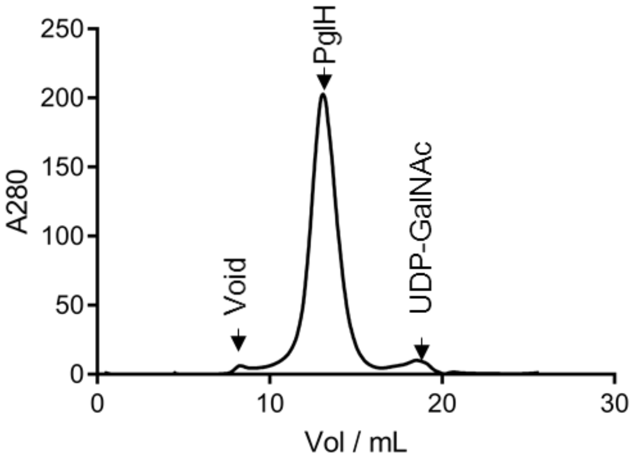

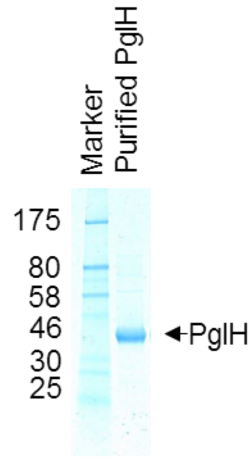

Supplementary Figure 2: PglH purification. Preparative size exclusion chromatography (SEC, left) and SDS-PAGE analysis (right) of purified PglH. Following affinity purification, PglH was concentrated in the presence of UDP-GalNAc and loaded on a Superdex S200 column at 0.5 mL/min. After preparative SEC, purified PglH was loaded on a 4-20% polyacrylamide gel. A single band is observed for the purified protein.

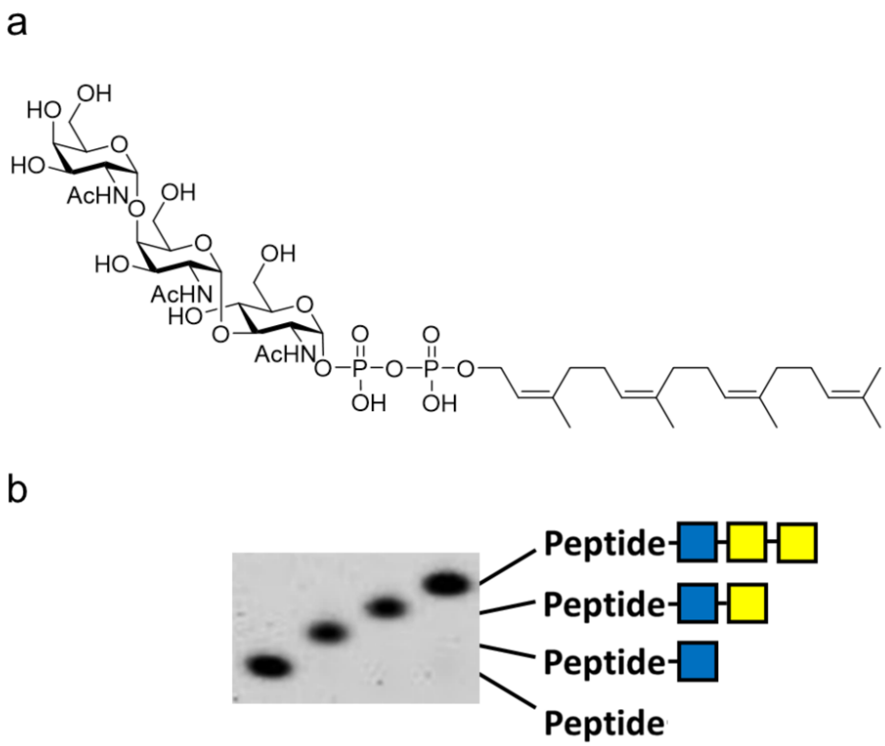

Supplementary Figure 3. Chemo-enzymatic synthesis of acceptor LLO substrate. (**a**) Chemical structure of the generated tri-LLO, GalNAc-α1,4-GalNAc-α1,3-GlcNAc-α1-PP-Neryl-neryl. To assess the completeness of the chemo-enzymatic synthesis, the glycans from the generated LLO analog were transferred onto a fluorescently labeled peptide using purified PglB protein. (**b**) Glycopeptides were subsequently analyzed by Tricine SDS–PAGE.

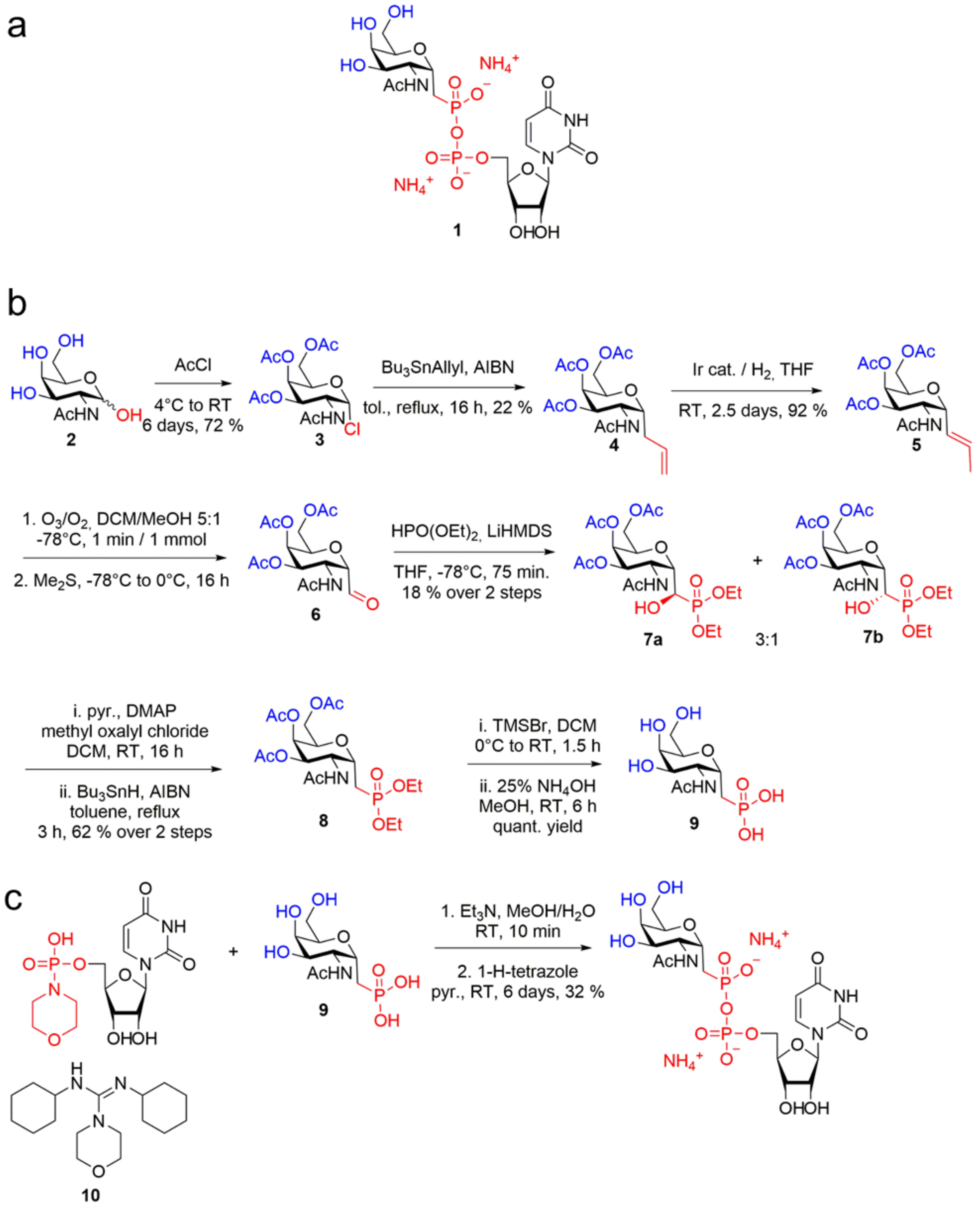

Supplementary Figure 4. Chemical synthesis of UDP-CH_2-GalNAc. (a) Chemical structure of UDP-CH_2-GalNAc. **(b)** Preparation of N-acetyl-D-galactosamine phosphonate precursor **9**. **(c)** Coupling reaction of phosphonate **9** and activated phosphate **10** to final pyrophosphate **1**.

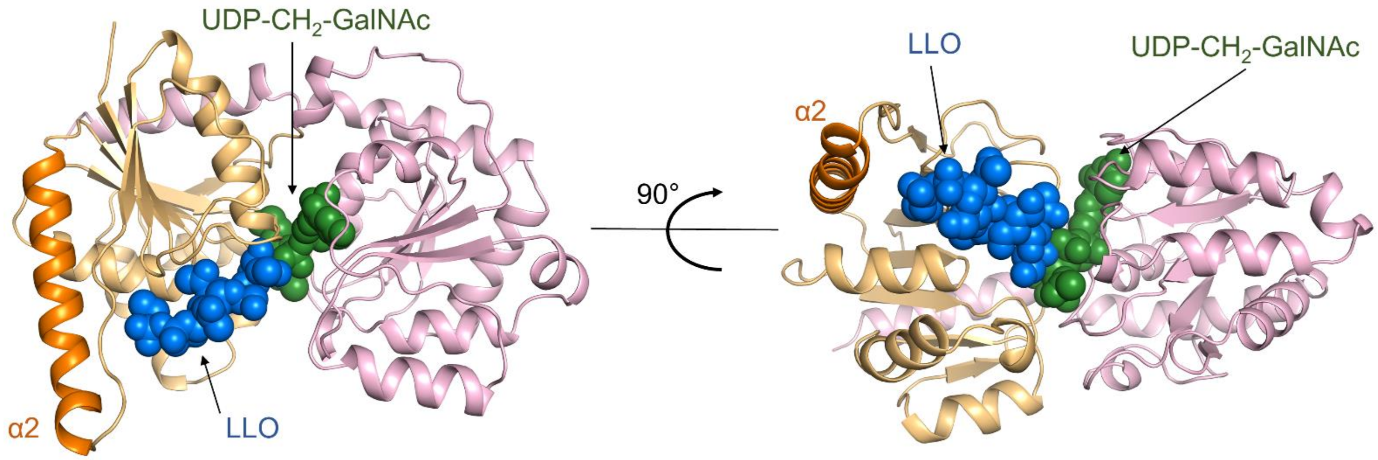

Supplementary Figure 5. Ribbon diagram of PglH in complex with synthetic LLO analog and UDP-CH_2-GalNAc, as shown in Fig. 2a but viewed from different angles. N-terminal and C-terminal domains are colored in light pink and light orange, respectively. Alpha helix α2 (termed "ruler helix") is shown in dark orange. Bound substrates are shown as spheres (green for UDP-CH_2-GalNAc, blue for LLO analog).

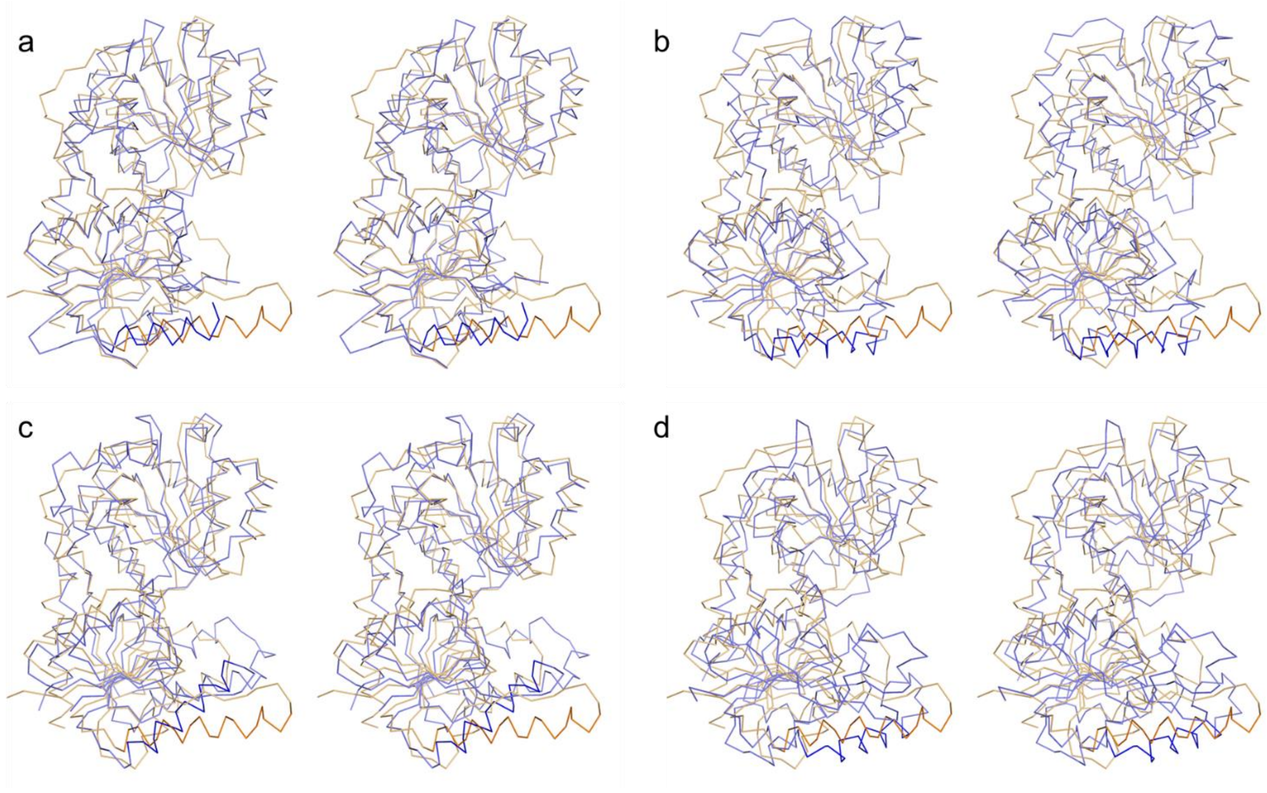

Supplementary Figure 6. Structural alignment of PglH with membrane associated GT-B glycosyltransferases. All panels show wall-eyed stereo views with backbone traces of PglH colored in light orange. GT-B glycosyltransferases superimposed onto PglH are shown in light blue. The ruler helix (α2) is colored in dark orange for PglH or dark blue for superimposed glycosyltransferases. **(a)** Superposition with phosphatidylinositol mannosyltransferase PimA from *M. smegmatis*, (RMSD: 2.23 for 266 Cα atoms). **(b)** Superposition with peptidoglycan glycosyltransferase MurG from *E. coli* (RMSD: 3.50 for 245 Cα atoms). **(c)** Superposition with lipopolysaccharide glucosyltransferase I WaaG from *E. coli* (RMSD: 2.32 for 296 Cα atoms). **(d)** Superposition with beta-glucuronosyltransferase GumK from *X. campestris* (RMSD: 2.89 for 227 Cα atoms).

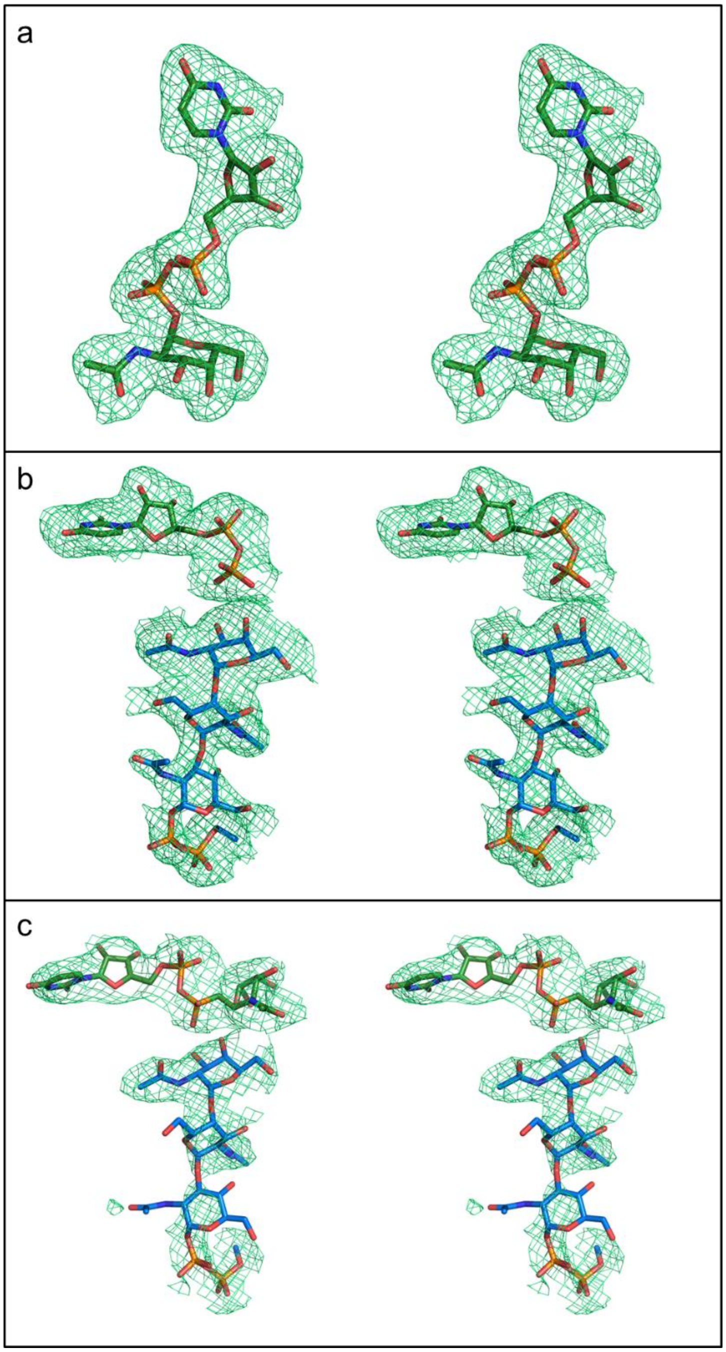

Supplementary Figure 7. Electron density maps of bound substrates. All panels show wall-eyed stereo views of polder omit maps contoured at 2.5σ, with the substrates shown as sticks. (**a**) Bound UDP-GalNAc of the final model of the structure at 2.3 Å resolution. **b**) Bound UDP and tri-LLO analog of the final model of the structure at 2.8 Å resolution. **c**) Bound UDP-CH2-GalNAc and tri-LLO analog of the final model of the structure at 3.3 Å resolution.

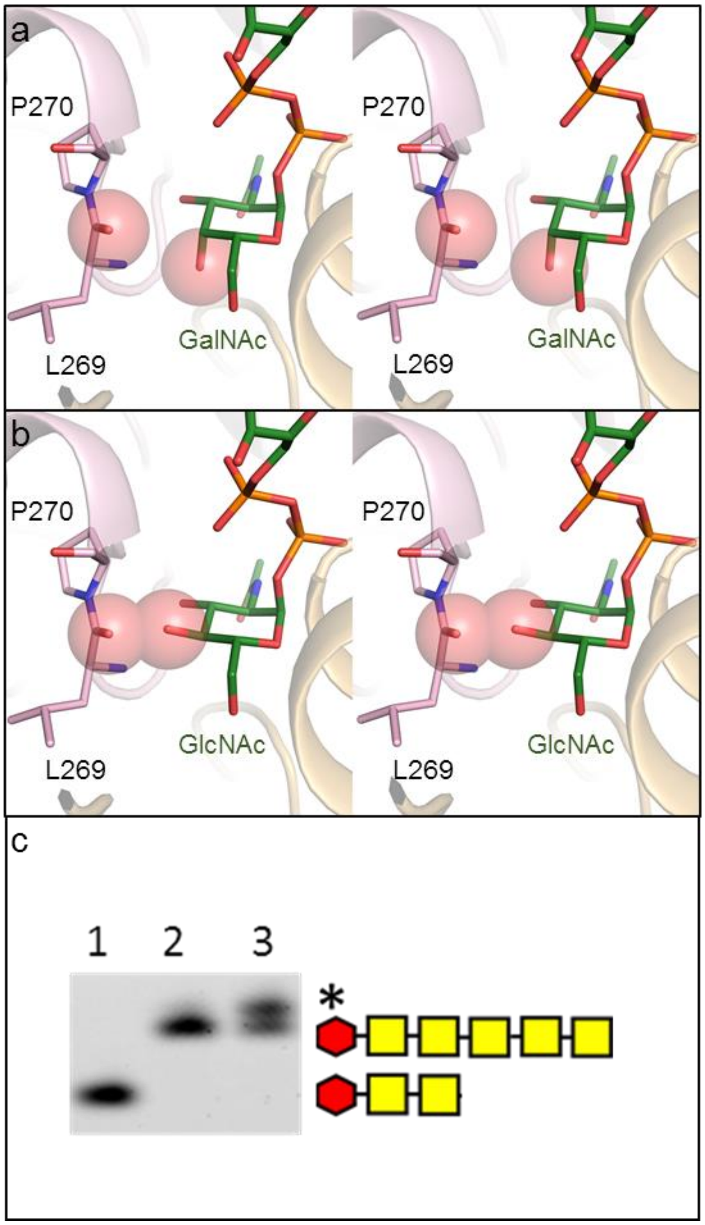

Supplementary Figure 8. Incompatibility of UDP-GlcNAc. (**a**) Stereo view of the binding site of PglH with bound UDP-GalNAc. The oxygen from the carbonyl group of the main chain of L269 and the oxygen in position 4’ of GalNAc are shown as red transparent van der Waals spheres. (**b**) Stereo view of the binding site of the donor substrate with a modeled UDP-GlcNAc molecule. The oxygen from the carbonyl group of the main chain of L269 and the oxygen in position 4’ of GlcNAc are shown as red transparent spheres and would produce a steric clash. **(c)** PglH is unable to transfer GlcNAc units. *In vitro* activity assays were performed using UDP-GlcNAc for 16h incubation (Lane 1), using UDP-GalNAc for 20 minutes incubation (Lane 2), and using UDP-GalNAc for 16 hours incubation (Lane 3). In lane three, an additional band is observed (asterisk), demonstrating the (very slow) transfer of a fourth GalNAc moiety.

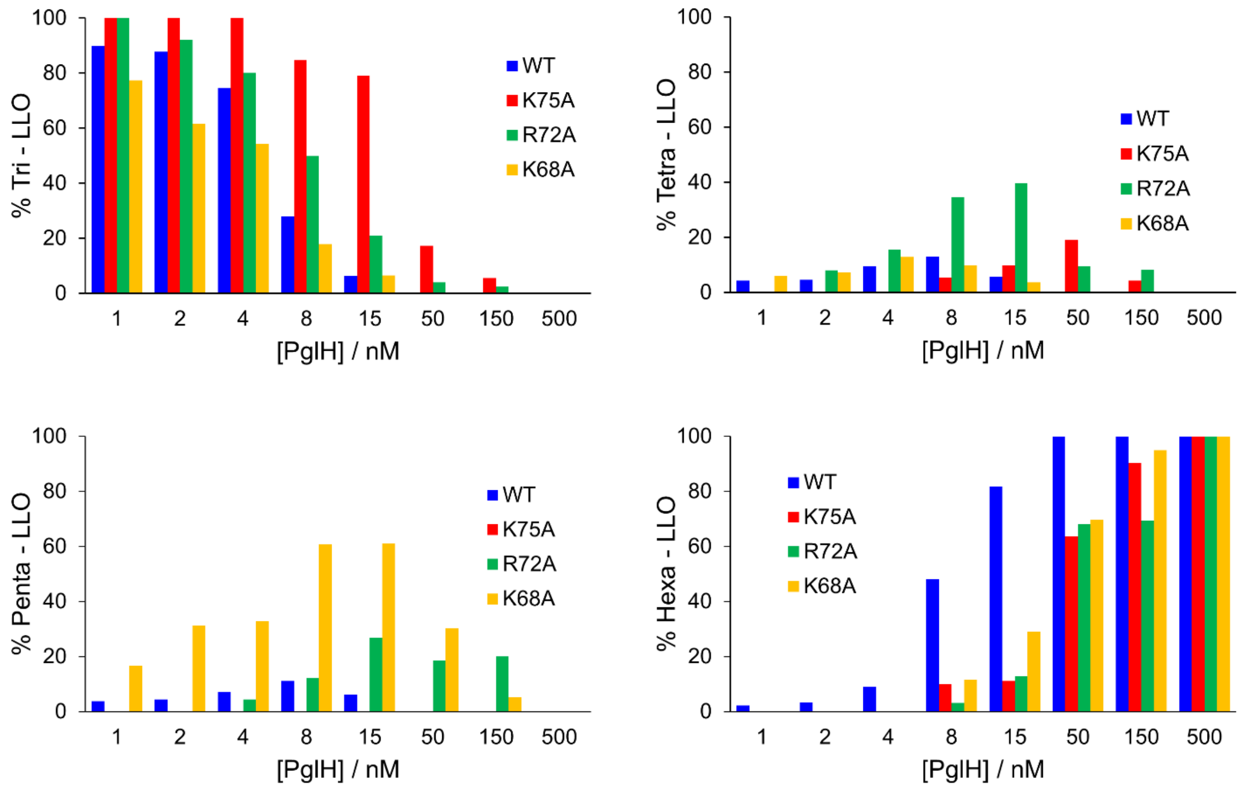

Supplementary Figure 9. Quantitation of LLO intermediates in PglH reactions *in vitro*. The relative ratios of substrate (Tri_LLO, top left panel), product (Hexa-LLO, bottom right panel), and the two intermediates (Tetra-LLO, top right panel; Penta-LLO, bottom left panel) were quantitated by analysis of the tricine SDS-PAGE analyses shown in Fig. 4b. Band intensities were measured using the program ImageJ and the percentage of each intermediate was calculated.

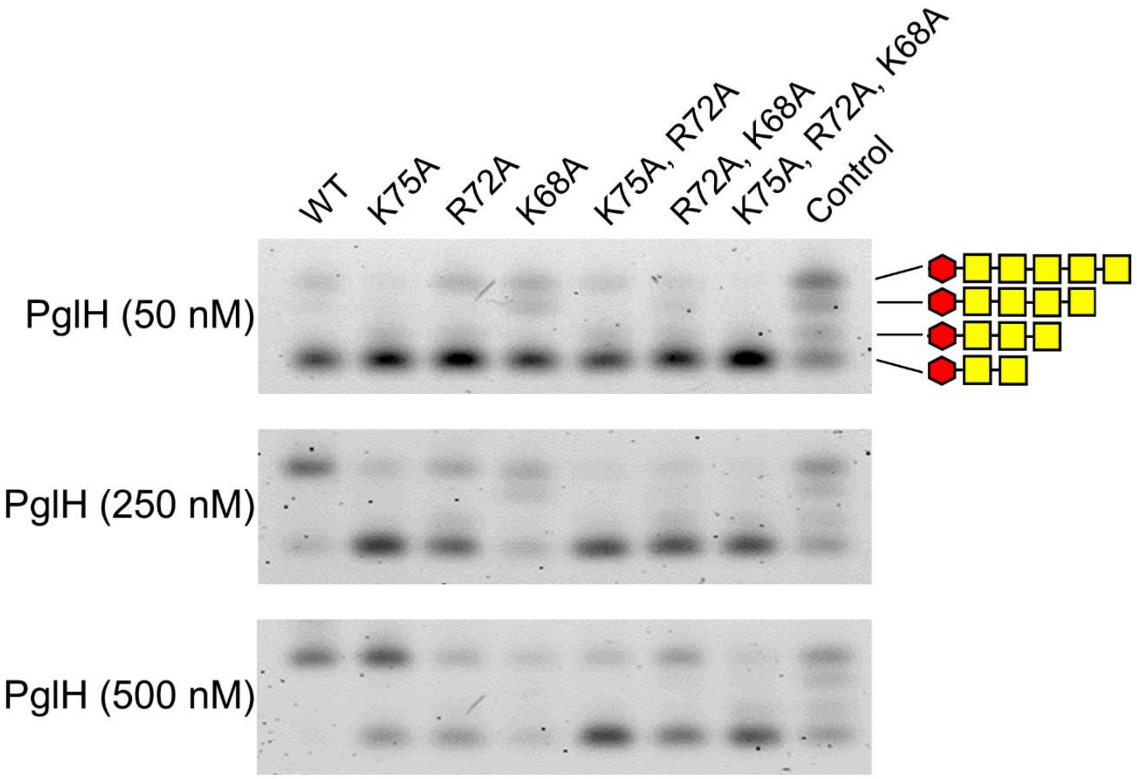

Supplementary Figure 10: Activity of wild type and mutant PglH on liposome-reconstituted, undecaprenyl-containing tri-LLO. Reactions were carried out for 10 minutes, after which they were stopped and the products used as donor substrates for *in vitro* glycosylation of fluorescently labeled peptide by PglB and analyzed by tricine SDS-PAGE, similar to the reactions shown in Fig. 4b. The amount of enzyme varied and is indicated on the left of each panel.

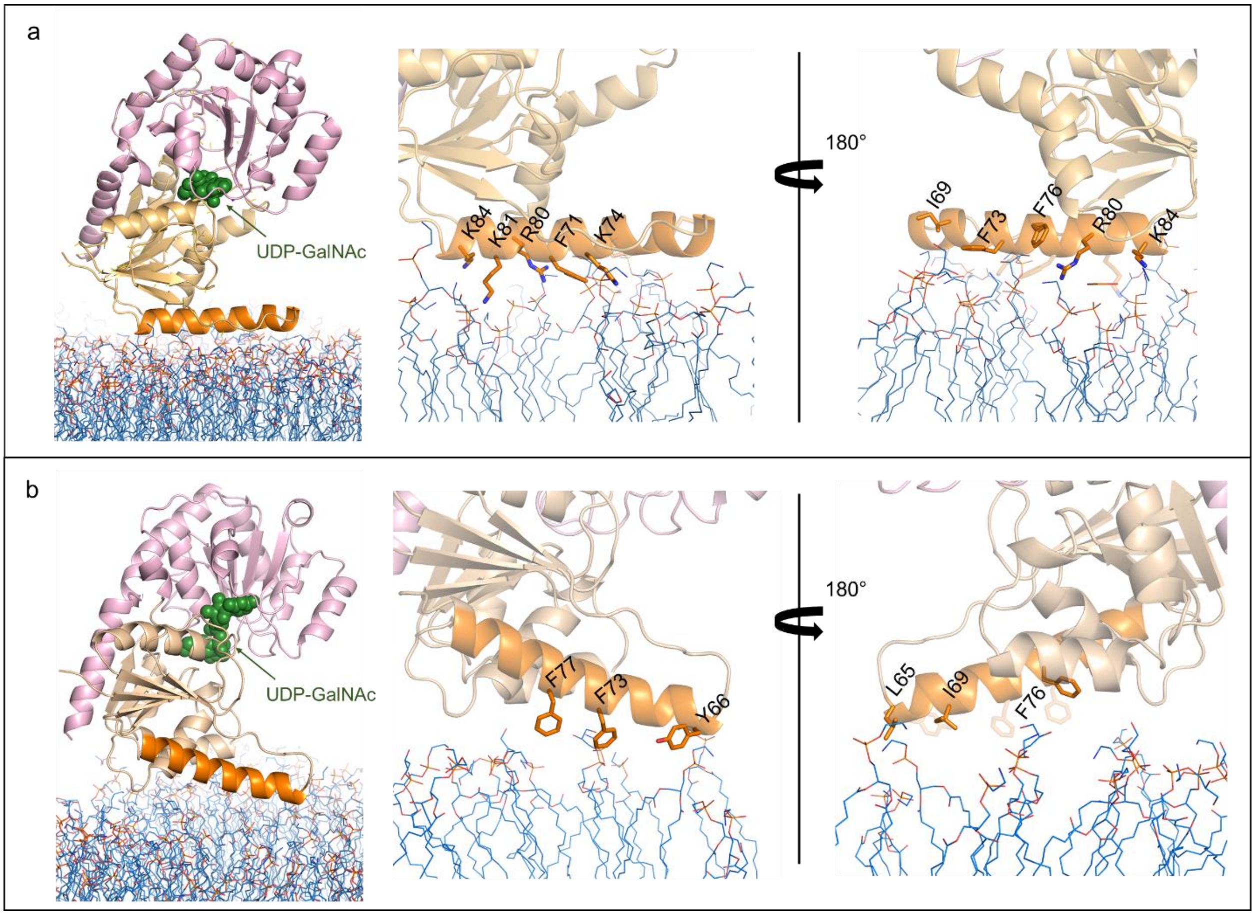

Supplementary Figure 11. Molecular dynamics simulations of PglH association with the lipid bilayer. MD simulations of PglH in complex with UDP-GalNAc and a lipid bilayer composed by 80% palmitoyl oleoyl phosphatidyl-ethanolamine (POPE) and 20% palmitoyl oleoyl phosphatidyl-glycerol (POPG), were performed. Two favorite modes of association of PglH with the lipid bilayer were found, as shown in **(a)** and **(b)**. In both panels, the donor substrate UDP-GalNAc is shown as green spheres and labeled; the ruler helix is colored in dark orange and close up views of its interaction with the lipid bilayer are shown. The relevant side chains of the ruler helix are labeled and represented as sticks.

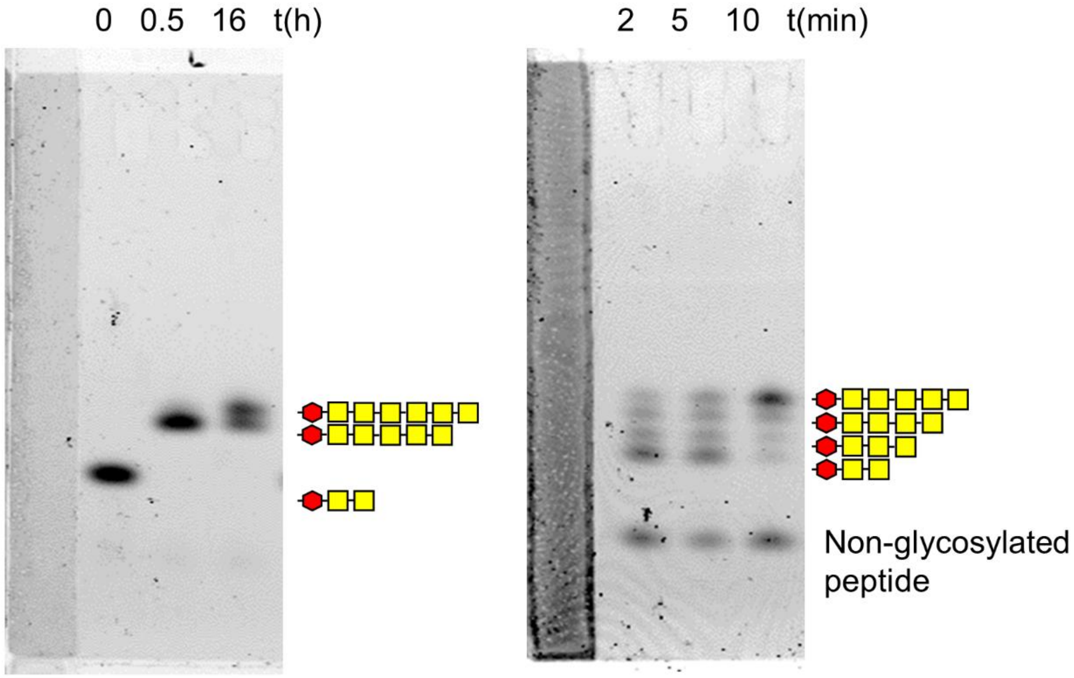

Supplementary Figure 12. Uncropped gel images from Fig. 1 b.

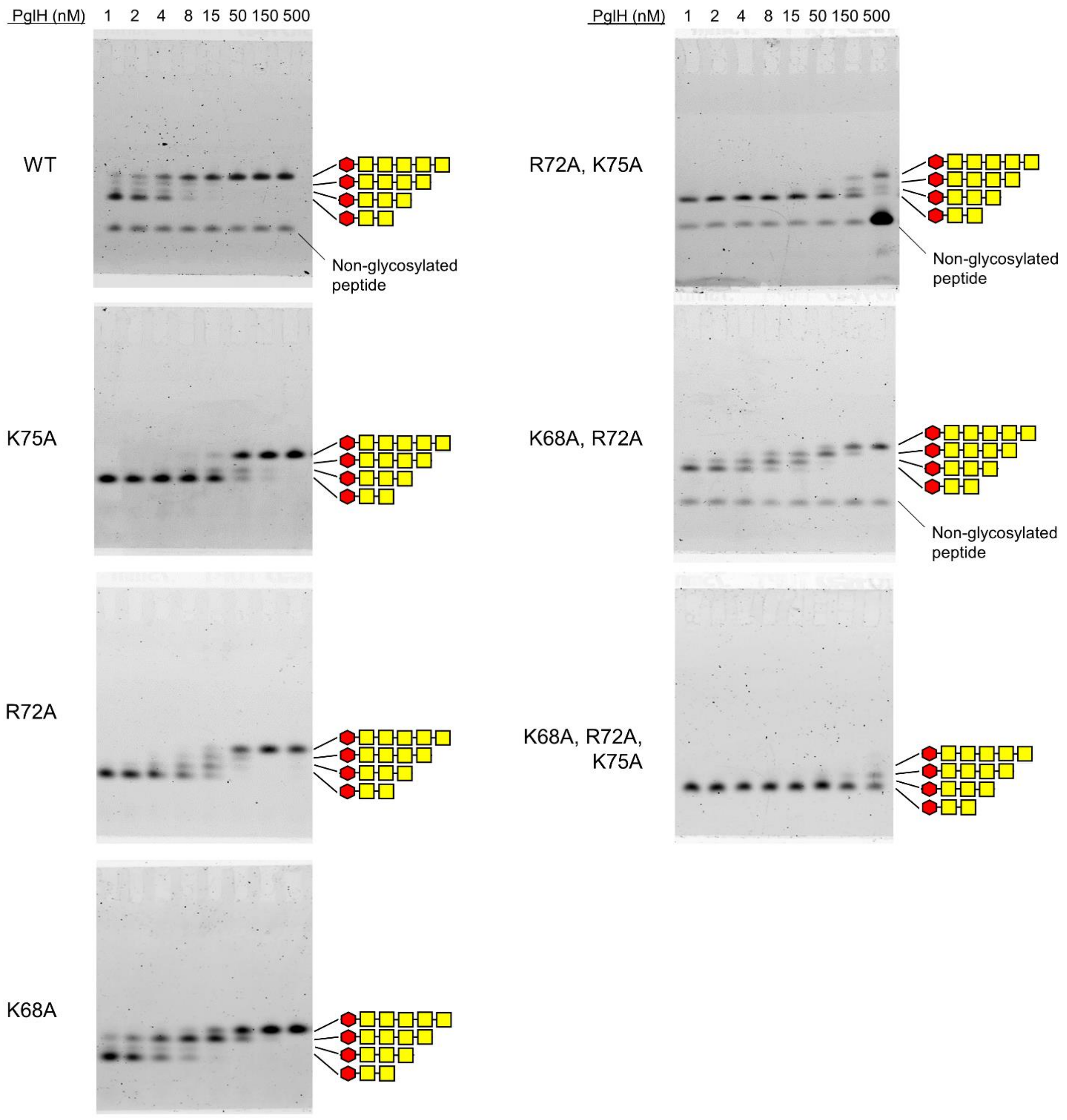

Supplementary Figure 13. Uncropped gel images from Fig. 4 b.

Supplementary Table 1. Statistics of native and SeMet SAD data collection and structure refinement

	PglH/UDP-GalNAc (SeMet)	PglH/UDP-GalNAc (SeMet)	PglH/LLO/UDP	PglH(SeMet)/LLO/ UDP-CH_2-GalNAc
**Data collection**				
Space group	P 1 21 1	P 1 21 1	P 1 21 1	P 1 21 1
Cell dimensions				
a, *b*, *c* (Å)	60.61, 125.13, 71.04	61.62, 127.03, 71.58	60.51, 125.78, 71.01	60.87, 126.91, 71.09
α, β, γ (°)	90, 90.247, 90	90, 90.134, 90	90, 90.285, 90	90, 90.265, 90
	Peak			
Wavelength (Å)	0.9787	1.0000	1.0000	1.0000
Resolution (Å)	50 – 2.5 (2.68 - 2.58)	29.94 - 2.3 (2.382 - 2.3)	29.69 - 2.7 (2.796 - 2.7)	29.78 - 3.3 (3.418 - 3.3)
R_{merge}	0.130 (1.497)	0.1571 (1.234)	0.2128 (1.743)	0.1772 (0.9563)
I / σI	11.7 (1.1)	11.49 (2.59)	8.95 (1.69)	8.33 (1.91)
Completeness (%)	97.4 (87.6)	98.2 (98.0)	98.0 (100)	99.8 (90.0)
Redundancy	6.9 (6.4)	6.8 (6.9)	6.7 (7.1)	6.7 (6.8)
**Refinement**				
Resolution (Å)		29.94 - 2.3 (2.382 - 2.3)	29.69 - 2.7 (2.796 - 2.7)	29.78 - 3.3 (3.418 - 3.3)
No. reflections		47981	28714	16099
R_{work} / R_{free}		0.1956 /0.2234	0.2067 / 0.2448	0.2171 / 0.2566
No. atoms				
Protein		5828	5828	5828
Ligand/ion		99	161	184
Water		203	45	0
B-factors				
Protein		39.64	55.82	68.75
Ligand/ion		37.73	58.11	112.39
Water		39.17	53.79	-
R.m.s. deviations				
Bond lengths (Å)		0.005	0.005	0.012
Bond angles (°)		0.86	0.85	1.35

Values shown correspond to statistics after anisotropy correction.
Values in parentheses are for highest-resolution shell.

Supplementary Table 2. Primers used for cloning of PglA and PglJ

Primer	Sequence (5' - 3')
FP_PglA	TACGCTATCATATGAGAATAGGATTTTTATCACATGCAGG
RP_PglA	TATATACTCGAGTCCTACATTCTTAATTACCCTATCATAAAG
FP_PglJ	TGTGTGTCATATGCAAAAATTAGGCATTTTTATTT
RP_PglJ	TATATATCTCGAGTGATCCTAATAAATATTTCAAAGC

Supplementary Methods

DNA sequence of the synthetic gene used for expression of PglH

ATGATGATGAAAATCTCCTTTATTATTGCCACCCTGAATTCGGGCGGAGCGGAACGC
GTCCTGGTCACACTGGCGAATGCCTTATGCAAGGAACATGAAGTCAGCATTATCAAG
TTTCATACTGGGGAGTCCTTCTACAAACTTGAGAACGAAGTGAAAGTAACGTCGTTG
GAGCAATTTCGCTTTGACACTCTGTACCACAAAATTGCTTCTCGTTTCAAAAAGTTTT
TTGCGTTGCGTAAAGCATTGAAAGAATCAAAAGCGGATGTATTCATCTCTTTTCTTG
ACACAACCAATATCGCTTGCATCCTTGCCAATATTGGCCTGAAAACCCCTTTAATTA
TCTCTGAACACTCAAATGAGGCATATTTAAAACCCAAGACGTGGCGCTTTCTTCGCC
GTGTATCGTATCCATTCTGCGACGCGCTGAGCGTCCTAGGATCATCAGATAAAGTTT
ACTATGAACGCTTTGTGAAGCGTGTGAAACTGCTTCTTAACCCGTGCCACTTCAGTG
ACGAGATTCCATTCGACAGCTCTTTCGAAAAAGAGAATCTGGTATTATTTATCGGAC
GCCTTGACCATAACAAGAACCCTGTGATGTTCTTAAAGGCTATCGCCCATCTGGATA
AAAATCTTCAGGAGAATTACAAATTTGTAATCGCTGGCGATGGGGAACTTCGTCAAG
AACTTGAGTATAAAGTTAAATCTTTGGGGATTAAGGTCGACTTCTTGGGCCGCGTTG
AAAATGTTAAGGCTTTGTATGAGAAGGCGAAGGTTCTTTGCCTTTGTAGTTTCGTTG
AGGGGCTTCCCACAGTTCTTATCGAAAGTTTATACTTCGAAGTTTGTCGCATCTCATC
TAGTTACTACAACGGAGCGAAGGACTTAATCAAAGATAATCACGACGGATTACTTG
TTGGCTGCGACGACGAAATTGCCCTTGCGAAAAAGCTTGAATTAGTGCTTAATGATG
AGAACTTCCGTAAAGAGTTGGTTAACAATGCGAAGCAACGTTGCAAGGATTTCGAA
ATTAGTAATATTAAAGAGGAATGGCTGAAATTGATCGTAGAGGTTAAGAATGCCTT
GGGATCCCATCATCACCATCATCACCATCATCACCATTAA

Protein sequence of the PglH construct expressed.

MMMKISFIIATLNSGGAERVLVTLANALCKEHEVSIIKFHTGESFYKLENEVKVTSLEQF
RFDTLYHKIASRFKKFFALRKALKESKADVFISFLDTTNIACILANIGLKTPLIISEHSNEA
YLKPKTWRFLRRVSYPFCDALSVLGSSDKVYYERFVKRVKLLLNPCHFSDEIPFDSSFEK
ENLVLFIGRLDHNKNPVMFLKAIAHLDKNLQENYKFVIAGDGELRQELEYKVKSLGIKV
DFLGRVENVKALYEKAKVLCLCSFVEGLPTVLIESLYFEVCRISSSYYNGAKDLIKDNHD
GLLVGCDDEIALAKKLELVLNDENFRKELVNNAKQRCKDFEISNIKEEWLKLIVEVKNA
LGSHHHHHHHHHH

Synthesis of UDP-CH_2-GalNAc

(2R,3R,4R,5R,6R)-5-acetamido-2-(acetoxymethyl)-6-chlorotetrahydro-2H-pyran-3,4-diyl diacetate (3)

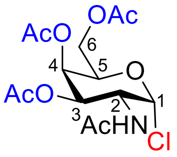

2 (14.00 g, 63.29 mmol, 1.00 eq) was suspended in acetyl chloride (27.00 ml, 379.73 mmol, 6.00 eq) at 4°C and 27 ml DCM was added. The reaction mixture was stirred at RT for 6 days under Ar. The resulting mixture was dissolved in 850 ml $CHCl_3$, washed with 3×850 ml ice water, 2×850 ml saturated $NaHCO_3$ and 850 ml brine, dried over Na_2SO_4 and concentrated to yield **3** (16.62 g, 45.44 mmol, 72 %) as a colorless foam which was used in the next step without further purifications (Rf = 0.13, DCM/MeOH 98:2). ^{1}H NMR (300 MHz, $CDCl_3$) δ = 6.26 (d, *J* = 3.6 Hz, 1H, H-1), 5.78 (d, *J* = 8.7 Hz, 1H, NH), 5.45 (d, *J* = 1.8 Hz, 1H, H-4), 5.27 (dd, *J* = 11.4 Hz, 3.3 Hz, 1H, H-3), 4.73-4.81 (ddd, *J* = 12.3 Hz, 9.0 Hz, 3.6 Hz, 1H, H-2), 4.47 (t, *J* = 6.6 Hz, 1H, H-5), 4.15 (d, *J* = 6.3 Hz, 1H, H-6a), 4.09 (d, *J* = 6.9 Hz, 1H, H-6b), 2.15 ‘s, 3H, NHAc), 2.04, 2.01, 1.99 (3xs, 3x3H, 3xOAc). ^{13}C NMR (75 MHz, $CDCl_3$) δ = 170.9, 170.4, 170.3, 170.0 (4xs, 3×OC=O, NHC=O), 95.0 (s, C-1), 69.9 (s, C-5), 67.4 (s, C-3), 66.6 (s, C-4), 61.2 (s, C-6), 49.3 (s, C-2), 23.2 (s, NHC=O$\underline{C}H_3$), 3×20.7 (3×s, 3×OC=O$\underline{C}H_3$).

(2R,3R,4R,5S,6R)-5-acetamido-2-(acetoxymethyl)-6-allyltetrahydro-2H-pyran-3,4-diyl diacetate (4)

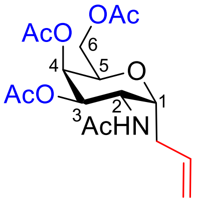

A suspension of **3** (16.35 g, 44.70 mmol, 1.00 eq), allyltributyltin (69.30 ml, 223.51 mmol, 5.00 eq) and AIBN (1.10 g, 6.71 mmol, 0.15 eq) in 90 ml dry toluene was stirred overnight at 90°C under an Ar atmosphere. The crude product was purified by flash column chromatography on silica gel (hexane/EtOAc 4:6 to 2:8 to 0:1). The residue was dissolved in 450 ml acetone and 45 ml of a 1% aqueous solution of HCl was added to the reaction mixture to convert the oxazoline into a more polar compound, (2R,3R,4R,5R,6S)-5-acetamido-2-(acetoxymethyl)-6-hydroxytetrahydro-2H-pyran-3,4-diyl diacetate. Acetone was removed and the residue was dissolved in DCM, washed with saturated $NaHCO_3$, brine, dried over Na_2SO_4, filtered and concentrated. The residue was purified by flash column chromatography on silica gel (hexane/EtOAc 2:8 to 0:1, Rf = 0.35, EtOAc) to yield **4** (3.70 g, 9.96 mmol, 22 %) as a colorless foam. ^{1}H NMR (300 MHz, $CDCl_3$) δ = 5.71-5.80 (m, 1H, H-8), 5.64 (d, *J* = 8.4 Hz, 1H, NH), 5.32-5.34 (m, 1H, H-4), 5.10-5.18 (m, 3H, H-3, H-9a, H-9b), 4.46-4.50 (m, 1H, H-2), 4.30-4.35 (m, 1H, H-1), 4.20-4.25 (m, 1H, H-6a), 4.09-4.13 (m, 1H, H-6b), 4.04-4.08 (m, 1H, H-5), 2.37-2.43 (m, 1H, H-7a), 2.23-2.29 (m, 1H, H-7b), 2.12, 2.07, 2.05, 1.98 (4xs, 4x3H, 3xOAc, NHAc). ^{13}C NMR (101 MHz, $CDCl_3$) δ = 171.0, 170.7, 170.2, 170.1 (4xs, 3×OC=O, NHC=O), 133.6 (s, C-8), 117.7 (s, C-9), 71.5 (s, C-1), 69.0 (s, C-5), 68.4 (s, C-3), 67.0 (s, C-4), 61.5 (s, C-6), 49.1 (s, C-2), 31.6 (s, C-7), 23.4 (s, NHC=O$\underline{C}H_3$), 21.0, 20.9, 20.8 (3×s, 3×OC=O$\underline{C}H_3$). ESI-MS (+) *m/z* calculated 372.17 (M+[H^+]), found 372.17 for $C_{17}H_{26}NO_8^+$.

(2R,3R,4R,5S,6R)-5-acetamido-2-(acetoxymethyl)-6-((E)-prop-1-en-1-yl)tetrahydro-2H-pyran-3,4-diyl diacetate (5)

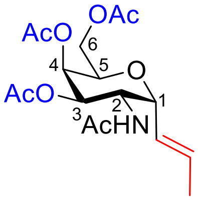

(1,5-Cyclooctadiene)bis(methyldiphenylphosphine)iridium(I) hexafluorophosphate (831 mg, 0.98 mmol, 0.10 eq) was dissolved in 100 ml THF and activated under an H_2 atmosphere until the opaque red suspension became a clear, slightly orange solution. Argon was then bubbled through the solution for 5 minutes to remove any H_2. The activated catalyst was then added to a solution of **4** (3.65 g, 9.83 mmol, 1.00 eq) in 90 ml THF and the reaction mixture was stirred at RT under an Ar atmosphere for 2.5 days. The orange mixture was concentrated under reduced pressure and the residue purified by flash column chromatography on silica gel (EtOAc/hexane 7:3 to 1:0, Rf = 0.35, EtOAc) to yield **5** (3.34 g, 8.99 mmol, 92 %) as a yellow solid. ^{1}H NMR (300 MHz, $CDCl_3$) δ = 5.82-5.92 (m, 1H, H-8), 5.61-5.67

(m, 1H, H-7), 5.48 (d, J = 8.4 Hz, 1H, NH), 5.34 (dd, J = 3.2 Hz, 1.6 Hz, 1H, H-4), 5.04 (dd, J = 10.8 Hz, 3.2 Hz, 1H, H-3), 4.55-4.64 (m, 2H, H-1, H-2), 4.07-4.13 (m, 3H, H-5, H-6a, H-6b), 2.14, 2.03, 2.02, 1.78 (4xs, 4x3H, 3xOAc, NHAc), 1.78 (d, 6.4 Hz, 3H, H-9). ^{13}C NMR (101 MHz, $CDCl_3$) δ = 171.4, 170.6, 170.4, 170.0 (4xs, 3×OC=O, NHC=O), 134.6 (s, C-8), 122.4 (s, C-7), 74.9 (s, C-1), 68.9 (s, C-3), 68.6 (s, C-5), 67.6 (s, C-4), 62.1 (s, C-6), 48.3 (s, C-2), 23.4 (s, NHC=O$\underline{C}H_3$), 20.9, 20.8, 20.8 (3×s, 3×OC=O$\underline{C}H_3$), 18.4 (s, C-9). ESI-HRMS (+) *m/z* calculated 372.1653 (M+[H^+]), found 372.1655 for $C_{17}H_{26}NO_8^+$.

(2R,3R,4R,5R,6S)-5-acetamido-2-(acetoxymethyl)-6-((diethoxyphosphoryl)(hydroxy)methyl)tetrahydro-2H-pyran-3,4-diyl diacetate (7a & 7b)

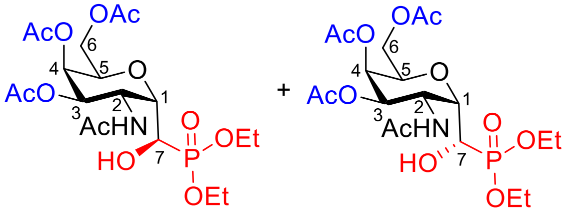

A steady stream of ozone in oxygen generated with an ozonizer was bubbled through a solution of **5** (2.53 g, 6.80 mmol, 1.00 eq) in 50 ml DCM/MeOH 4:1 for 30 min at -78°C. Then, the color of the solution turned blue, meaning an excess of ozone and a total conversion of the reaction. The reaction mixture was stirred under an Ar atmosphere at -78°C for 10 min. A steady stream of oxygen was bubbled through the reaction mixture for 10 min to remove the excess of ozone in the solution. Under an Ar atmosphere, dimethyl sulfide (2.51 ml, 34.00 mmol, 5.00 eq) was added to the reaction mixture which was allowed to warm up to RT overnight. The reaction mixture was concentrated under reduced pressure. The aldehyde **6** (colorless foam) was used in the next step without further purification. To a solution of diethyl phosphite (1.13 g, 8.16 mmol, 1.20 eq) in 45 ml THF was added LiHMDS (8.16 ml, 8.16 mmol, 1.20 eq, 1 M in THF) dropwise at -78°C. After 15 min, a solution of the aldehyde (2.44 g, 6.80 mmol, 1.00 eq) in 15 ml THF was added and the resulting mixture was stirred at -78°C for 1h30. The reaction was quenched by addition of 25 ml saturated NH_4Cl and 260 ml Et_2O. The reaction mixture was stirred 15 min at -78°C and was then warmed up to RT. 35 ml of water was added and the organic layer was separated. The aqeous layer was extracted with 2x85 ml diethyl ether and the combined organic fractions were dried over Na_2SO_4, filtered and concentrated. The residue was purified by flash column chromatography on silica gel (DCM/MeOH 98:2 to 95:5) to yield a major diastereoisomer (447 mg, 0.90 mmol, 13 %, Rf = 0.31, DCM/MeOH 94:6) and a minor diastereoisomer (145 mg, 0.29 mmol, 4 %, Rf = 0.27, DCM/MeOH 94:6) as colorless foams. ^{1}H NMR major diastereoisomer (400 MHz, $CDCl_3$) δ = 7.09 (d, J = 8.4 Hz, 1H, NH), 5.47 (dd, J = 8.0 Hz, 3.6 Hz, 1H), 5.37 (t, J = 3.0 Hz, 1H), 4.53-4.59 (m, 1H, H-2), 4.39-4.45 (m, 2H, H-5, H-6a), 4.30-4.34 (m, 1H), 4.12-4.19 (m, 5H, 2xOC$\underline{H_2}$CH$_3$), 4.07 (dd, J = 11.6 Hz, 4.4 Hz, 1H, H-6b), 2.09, 2.05, 2.05, 2.01 (4xs, 4x3H, 3xOAc, NHAc), 1.31-1.36 (m, 6H, 2xOCH$_2$C$\underline{H_3}$). ^{13}C NMR major diastereoisomer (101 MHz, $CDCl_3$) δ = 171.5, 171.0, 170.2, 170.1 (4xs, 3×OC=O, NHC=O), 71.7 (s, C-5), 70.7 (s), 69.2 (s), 68.7 (s), 66.5 (s), 63.4, 63.3 (2xd, J = 2x7.1 Hz, 2xO$\underline{C}$H$_2$CH$_3$), 60.9 (s, C-6), 48.1 (d, J = 4.9 Hz, C-2), 23.3 (s, NHC=O$\underline{C}H_3$), 21.0, 20.9, 20.8 (3×s, 3×OC=O$\underline{C}H_3$), 16.6, 16.5 (2xs, 2x OCH$_2\underline{C}$H$_3$). ^{31}P NMR major diastereoisomer (122 MHz, $CDCl_3$) δ = 22.2. ESI-HRMS major diastereoisomer (+) *m/z* calculated 498.1735 (M+[H^+]), found 498.1732 for $C_{19}H_{33}NO_{12}P^+$. ^{1}H NMR minor diastereoisomer (400 MHz, $CDCl_3$) δ = 6.66 (d, J = 8.8 Hz, 1H, NH), 5.74 (dd, J = 11.2 Hz, 3.6 Hz, 1H), 5.36-5.40 (m, 1H), 4.70-4.77 (m, 1H), 4.50-4.56 (m, 1H), 4.40-4.45 (m, 1H), 4.28-4.37 (m, 2H), 4.07-4.20 (m, 4H, 2xOC$\underline{H_2}$CH$_3$), 3.96-4.01 (m, 1H), 2.13, 2.01, 2.01, 1.99 (4xs, 4x3H, 3xOAc, NHAc), 1.28-1.38 (m, 6H, 2xOCH$_2$C$\underline{H_3}$). ^{13}C NMR minor diastereoisomer (101 MHz, $CDCl_3$) δ = 171.0, 171.0, 170.5, 170.5 (4xs, 3×OC=O, NHC=O), 73.7 (s), 71.1 (s), 70.9 (s), 69.5 (s), 67.1 (s), 2x65.1 (2xd, J = 2x7.1 Hz, 2xO$\underline{C}$H$_2$CH$_3$), 61.3 (s, C-6), 47.2 (d, J = 10.9 Hz, C-2), 23.2 (s, NHC=O$\underline{C}H_3$), 21.0, 20.9, 20.8 (3×s, 3×OC=O$\underline{C}H_3$), 16.6, 16.5 (2xs, 2x OCH$_2\underline{C}$H$_3$). ^{31}P NMR major diastereoisomer (122 MHz, $CDCl_3$) δ = 23.4. ESI-HRMS major diastereoisomer (+) *m/z* calculated 498.1735 (M+[H^+]), found 498.1733 for $C_{19}H_{33}NO_{12}P^+$.

(2R,3R,4R,5R,6S)-5-acetamido-2-(acetoxymethyl)-6-((diethoxyphosphoryl)methyl)tetrahydro-2H-pyran-3,4-diyl diacetate (8)

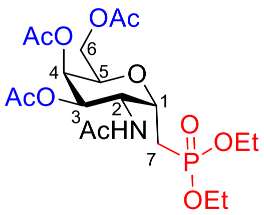

To a solution of **7** (107 mg, 0.22 mmol, 1.00 eq), 4 Å crushed molecular sieves in 4.5 ml dry DCM was added pyridine (0.17 ml, 2.15 mmol, 10.00 eq), DMAP (52.6 mg, 0.43 mmol, 2.00 eq) and methyl oxalyl chloride (0.10 ml, 1.08 mmol, 5.00 eq) respectively. The reaction mixture was stirred at RT under an Ar atmosphere overnight. The reaction mixture was filtered on celite, acidified to pH 1 with 1M HCl and distilled water was added. The phases were separated and the aqueous phase was extracted with DCM. The combined organic phases were washed with brine, dried over Na_2SO_4, filtered and concentrated to dryness. The residue was dissolved 1.9 ml toluene and Bu_3SnH (0.29 ml, 1.08 mmol, 5.00 eq) and AIBN (5.30 mg, 0.03 mmol, 0.15 eq) were added. The reaction mixture was heated to reflux and stirred for 3h and purified by flash column chromatography on silica gel (DCM/MeOH 98:2 to 96:4, Rf = 0.32, DCM/MeOH 96:4) to yield **8** (64 mg, 0.13 mmol, 62 %) as a colorless foam. ^{1}H NMR (300 MHz, $CDCl_3$) δ = 6.49 (d, *J* = 8.4 Hz, 1H, NH), 5.35 (t, *J* = 2.8 Hz, 1H), 5.11 (dd, *J* = 10.0 Hz, 2.4 Hz, 1H), 4.51-4.65 (m, 2H), 4.19-4.22 (m, 1H), 4.08-4.16 (m, 6H, 2xOC$\underline{H_2}$$CH_3$), 2.11, 2.04, 2.03, 1.98 (4xs, 4x3H, 3xOAc, NHAc), 1.29-1.34 (m, 6H, 2xOCH_2C$\underline{H_3}$). ^{13}C NMR (101 MHz, $CDCl_3$) δ = 169.7, 169.5, 169.5, 169.1 (4xs, 3×OC=O, NHC=O), 68.2 (s), 67.0 (s), 65.6 (s), 61.5 (d, *J* = 6.3 Hz, O$\underline{C}$$H_2CH_3$), 61.0 (d, *J* = 6.7 Hz, O$\underline{C}$$H_2CH_3$), 59.9 (s), 47.6 (d, *J* = 8.8 Hz, C-2), 26.3 (d, *J* = 10.6 Hz), 22.2 (s, NHC=O$\underline{C}$$H_3$), 19.8, 19.7, 19.7 (3×s, 3×OC=O$\underline{C}$$H_3$), 15.5, 15.4 (2xs, 2xOC$H_2$$\underline{C}$$H_3$). ^{31}P NMR (122 MHz, $CDCl_3$) δ = 27.5. ESI-HRMS (+) *m/z* calculated 482.1786 (M+[H^+]), found 482.1789 for $C_{19}H_{33}NO_{11}P^+$.

(((2S,3R,4R,5R,6R)-3-acetamido-4,5-dihydroxy-6-(hydroxymethyl)tetrahydro-2H-pyran-2-yl)methyl)phosphonic acid (9)

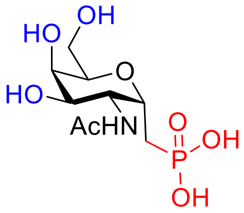

8 (95 mg, 0.20 mmol, 1.00 eq) was dissolved in 1.5 ml DCM. At 0°C under an Ar atmosphere, TMSBr (0.52 ml, 3.95 mmol, 20.00 eq) was added dropwise to the reaction mixture which was then stirred at RT for 1.5 h. Another 20 eq TMSBr were added to the reaction mixture which was then stirred for another 1.5 h. The reaction mixture was concentrated under reduced pressure, dissolved in 5 ml acetone and 0.13 ml water. The mixture was stirred for 30 min and then concentrated *in vacuo*. The residue was dissolved in 5 ml MeOH and NH_4OH (9.43 ml, 59.2 mmol, 300 eq) was added. The reaction mixture was stirred at RT overnight and freeze-dried to yield **9** (59 mg, 0.20 mmol, quantitative yield) as a beige powder which was used in the next step without further purification.

(((2S,3R,4R,5R,6R)-3-acetamido-4,5-dihydroxy-6-(hydroxymethyl)tetrahydro-2H-pyran-2-yl)methyl)phosphonic (((2R,3S,4R,5R)-5-(2,4-dioxo-3,4-dihydropyrimidin-1(2H)-yl)-3,4-dihydroxytetrahydrofuran-2-yl)methyl phosphoric) anhydride (1)

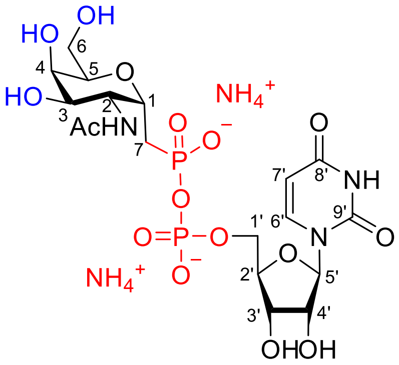

9 (59 mg, 0.20 mmol, 1.00 eq) was dissolved in 4 ml MeOH and 0.3 ml H_2O, treated with 0.15 ml Et_3N, stirred at RT for 10 min and concentrated to dryness. The residue was then co-evaporated 3 times with dry pyridine. 1-H-tetrazole (55.3 mg, 0.79 mmol, 4.00 eq, 0.45 M in MeCN) was dried with a flux of Ar, dissolved in 0.85 ml pyridine and added to **10** (406 mg, 0.59 mmol, 3.00 eq). This reaction mixture was then added to a solution of **9** dissolved in 0.85 ml pyridine and the reaction mixture was stirred at RT under an Ar atmosphere for 6 days. The reaction mixture was concentrated to dryness. The residue was split into 2 batches and each of them were dissolved in 2 ml of a 0.25 M NH_4HCO_3 buffer and applied to size exclusion chromatography (Bio-Rad, Bio-Gel, P-2 fine, 2.2 x 100 cm) eluted with the previous buffer. Pure fractions according to HRMS were freeze-dried.
The combined fractions of both batches were applied one more time to size exclusion chromatography and

pure fractions according to HRMS were freeze-dried to yield **1** (40 mg, 0.06 mmol, 32 %) as a colorless lyophilisat. ^{1}H NMR (400 MHz, MeOD) δ = 8.04 (d, *J* = 8.0 Hz, 1H, H-6'), 5.94 (d, *J* = 5.2 Hz, 1H, H-5'), 5.83 (d, *J* = 8.0 Hz, 1H, H-7'), 4.50-4.57 (m, 1H, H-1), 4.20-4.34 (m, 5H, H-2, H-5, H-1'a, H-1'b, H-4'), 4.12 (dd, *J* = 4.4 Hz, 2.4 Hz, 1H, H-2'), 3.96-3.99 (m, 1H, H-3'), 3.86-3.91 (m, 2H, H-4, H-6a), 3.77 (dd, *J* = 9.6 Hz, 3.2 Hz, 1H, H-3), 3.65 (dd, *J* = 11.6 Hz, 3.2 Hz, 1H, H-3), 2.29-2.40 (m, 1H, H-7a), 1.99 (s, 3H, NHAc), 1.86-1.96 (m, 1H, H-7b). ^{13}C NMR (101 MHz, MeOD) δ = 172.5 (s, NH$\underline{C}$=OCH_3), 164.9 (s, C-8'), 151.2 (s, C-9'), 141.4 (s, C-6'), 101.7 (s, C-7'), 88.6 (s, C-5'), 83.6 (d, *J* = 9.2 Hz, C-2'), 74.3 (s, C-4'), 72.8 (s, C-3'), 69.7 (s, C-5), 69.3 (d, *J* = 2.5 Hz, C-1), 69.2 (s, C-4), 68.4 (s, C-3), 64.5 (d, *J* = 5.4 Hz, C-1), 61.3 (s, C-6), 50.7 (d, *J* = 12.4 Hz, C-2), 26.0 (d, *J* = 140.2 Hz, C-7), 21.4 (s, NHC=O$\underline{C}H_3$). ^{31}P NMR (122 MHz, MeOD) δ = 13.6 (d, *J* = 25.4 Hz, P1), -10.9 (d, *J* = 25.4 Hz, P2). ESI-HRMS (-) *m/z* calculated 604.0950 (M-[H^+]), found 604.0949 for $C_{18}H_{28}N_3O_{16}P_2^-$.
